# Engineering a Rapid Insulin Release System Controlled By Oral Drug Administration

**DOI:** 10.1002/advs.202105619

**Published:** 2022-01-20

**Authors:** Mohamed Mahameed, Shuai Xue, Bozhidar‐Adrian Stefanov, Ghislaine Charpin‐El Hamri, Martin Fussenegger

**Affiliations:** ^1^ Department of Biosystems Science and Engineering ETH Zurich Mattenstrasse 26 Basel CH‐4058 Switzerland; ^2^ Département Génie Biologique Institut Universitaire de Technologie Université Claude Bernard Lyon 1 Villeurbanne Cedex F‐69622 France; ^3^ University of Basel Faculty of Life Science Basel CH‐4058 Switzerland

**Keywords:** diabetes, endoplasmic reticulum, insulin, rapid release

## Abstract

Rapid insulin release plays an essential role in maintaining blood‐glucose homeostasis in mammalians. Patients diagnosed with type‐I diabetes mellitus experience chronic and remarkably high blood‐sugar levels, and require lifelong insulin injection therapy, so there is a need for more convenient and less invasive insulin delivery systems to increase patients’ compliance and also to enhance their quality of life. Here, an endoplasmic‐reticulum‐localized split sec‐tobacco etch virus protease (TEVp)‐based rapamycin‐actuated protein‐induction device (RAPID) is engineered, which is composed of the rapamycin‐inducible dimerization domains FK506 binding protein (FKBP) and FKBP‐rapamycin binding protein fused with modified split sec‐TEVp components. Insulin accumulation inside the endoplasmic reticulum (ER) is achieved through tagging its C‐terminus with KDEL, an ER‐retention signal, spaced by a TEVp cleavage site. In the presence of rapamycin, the split sec‐TEVp‐based RAPID components dimerize, regain their proteolytic activity, and remove the KDEL retention signal from insulin. This leads to rapid secretion of accumulated insulin from cells within few minutes. Using liver hydrodynamic transfection methodology, it is shown that RAPID quickly restores glucose homeostasis in type‐1‐diabetic (T1DM) mice treated with an oral dose of clinically licensed rapamycin. This rapid‐release technology may become the foundation for other cell‐based therapies requiring instantaneous biopharmaceutical availability.

## Graphical abstract

The split sec‐tobacco etch virus protease (TEVp)‐based rapamycin‐actuated protein‐induction device (RAPID) localized in the endoplasmic reticulum represents a universal protein fast‐release platform. In the presence of rapamycin, the ss‐TEVp parts dimerize, regain proteolytic activity, and remove the KDEL retention signal from the protein of interest (POI), which enables rapid release of the POI within minutes.

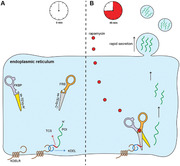

## 1 Introduction

Insulin is a peptide hormone produced, stored, and vesicularly released by glucose‐sensing pancreatic *β*‐cells as a response to elevated blood sugar levels.^[^ ^1^ ^]^ Once released into the bloodstream, insulin promotes glucose uptake into peripheral tissues, thereby maintaining blood‐glucose levels in the normoglycemic range.^[^ ^2^ ^]^ In order to maintain glucose homeostasis, insulin is rapidly secreted in response to an increase in blood sugar concentration.^[^ ^3^, ^4^ ^]^ Type‐1 diabetes mellitus (T1DM) is an endocrine medical condition in which pancreatic *β*‐cells are destroyed and thus no longer able to produce sufficient insulin to maintain glucose homeostasis.^[^ ^5^ ^]^ Patients with T1DM experience permanently high blood glucose levels, which lead to fatal complications if left untreated by the supplementation of external insulin.^[^ ^6^ ^]^ Thus, exogenous insulin is generally delivered to diabetic patients via injection several times a day.^[^ ^7^ ^]^ Repetitive, site‐confined insulin injections have a detrimental influence on T1DM patients’ compliance and quality of life.^[^ ^8^, ^9^ ^]^ Furthermore, regular insulin injections are associated with multiple side effects, particularly hypoglycemia and weight gain.^[^ ^10^ ^]^ In recent years, there have been many attempts to develop orally active insulin, but so far without great success.^[^ ^11^ ^]^

TEVp is a site‐specific peptidase that has become a valuable biochemical tool to cleave certain peptide bonds both in vitro and cellular systems.^[^ ^12^ ^]^ TEVp recognizes a linear epitope of seven amino acid residues (ENLYFQ‐G/S).^[^ ^13^ ^]^ The need for tunable protease activity led to the development of a split version of TEVp,^[^ ^14^ ^]^ which is composed of two separate parts of the catalytic region that regain their proteolytic activity upon juxtaposition. Neither intact TEVp nor its split variants are functional in the secretory pathway of mammalian cells, as each undergoes multiple posttranslational modifications that disrupt the proteolytic activity.^[^ ^15^ ^]^ Mutagenesis‐based screening led to the development of a TEVp version called sec‐TEVp, which remains active in the secretory compartments and cleave target proteins in the endoplasmic reticulum (ER), Golgi apparatus, and secretory vesicles.^[^ ^15^ ^]^

Here, we engineered an ER‐localized split sec‐TEVp‐based rapamycin‐actuated protein‐induction device (RAPID), which enables rapamycin‐adjustable cleavage and release of secretory proteins engineered to be retained in the ER. We first engineered split versions of sec‐TEVp containing FKBP or FRB domains for rapamycin‐inducible heterodimerization,^[^ ^16^ ^]^ secretion signals to direct them into the secretory pathway,^[^ ^17^ ^]^ and the ER retention signal KDEL to associate them with ER‐located KDEL receptors.^[^ ^18^, ^19^ ^]^ To design a rapid‐release system for insulin mimicking vesicular secretion of pancreatic *β*‐cells, we constructed an ER‐retained version of insulin containing TEVp cleavage site (TCS; ENLYFQ/S) between the insulin *α*‐chain and the KDEL signal. Upon addition of rapamycin, the RAPID components heterodimerize, thereby restoring their proteolytic activity, cleaving the TCS and removing the KDEL retention signal which enables rapid secretion of accumulated insulin from the cell within few minutes.

## 2 Results

**Figure 1:**
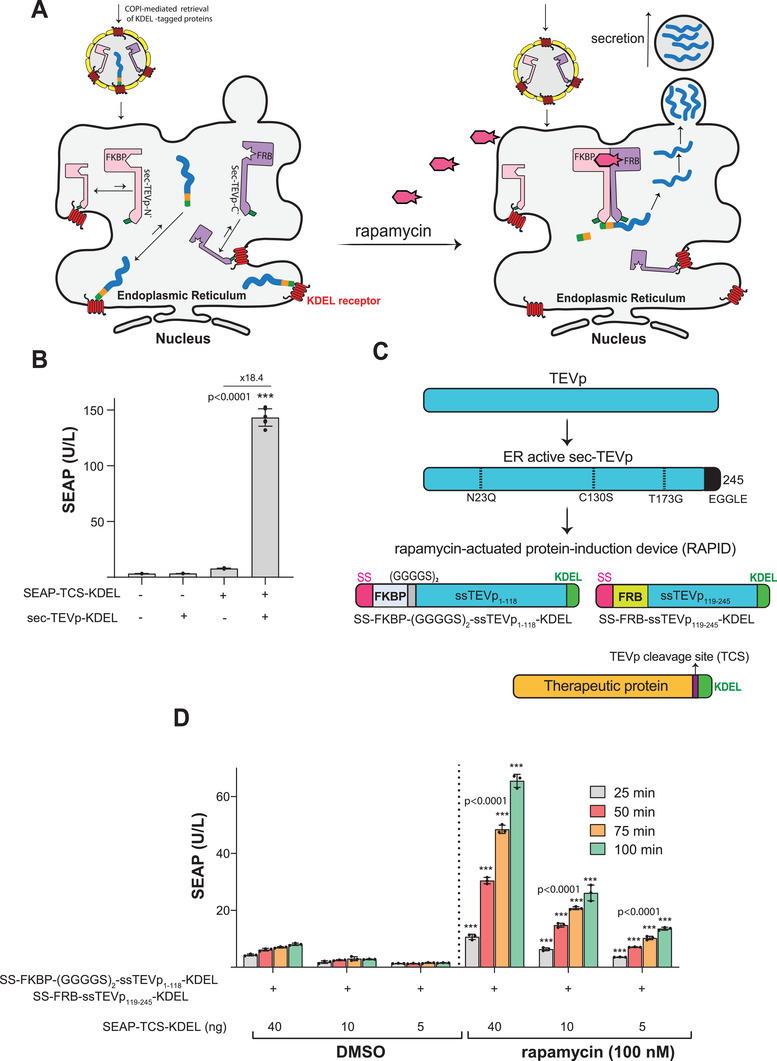
Design of fast‐release system using rapamycin as a signal input. A) Schematic illustration of RAPID in mammalian cells. The protein of interest (shown in blue) is C′‐terminally fused with a TEVp cleavage site (TCS) (shown in orange) and a KDEL retention signal (shown in green). In the absence of rapamycin, the system components are accumulated inside the ER through interaction with KDEL receptor (shown in red). Once rapamycin is added, RAPID components dimerize and regain catalytic functionality, cleaving the KDEL retention signal, and allowing the protein of interest to be secreted into the extracellular space. RAPID components are retained in the ER, as they contain an uncleavable KDEL signal that prevents their secretion. If KDEL‐bearing proteins escape the ER, they would be translocated back from the Golgi complex to the ER through COPI‐mediated retrieval vesicles (yellow). B) SEAP levels after 24 h in culture supernatants of HEK‐293 cells transfected with pMMH10 and pMMH51 plasmids, which encode SEAP‐TCS‐KDEL (P_hCMV_‐SEAP‐TCS‐KDEL‐pA) and intact sec‐TEVp‐KDEL (P_hCMV_‐sec‐TEVp‐KDEL‐pA) separately or in combination at a 1:1 ratio (50 ng each). Data are presented as means ± s.d. of *n* = 6 biologically independent samples. ^***^ *p* < 0.001. C) Schematic illustration of the protein components of the RAPID expression system. Sec‐TEVp was split into two parts; sec‐TEVp‐N′_1–118_ and sec‐TEVp‐C′_119–245_, which are fused with FKBP and FRB, respectively. Both constructs are tagged with a C′‐terminal KDEL retention signal and an N′‐terminal calreticulin secretion signal (SS), which directs them to the ER. To enhance the dimerization efficiency, a flexible linker (GGGGS)_2_ is inserted between FKBP and sec‐TEVp‐N′_1–118_. D) SEAP levels in culture supernatants of HEK‐293 cells transfected with pMMH26 and PMMH27 plasmids, which encode SS‐FKBP‐(GGGGS)_2_‐ssTEVp_1–118_‐KDEL (P_hCMV_‐SS‐FKBP‐(GGGGS)_2_‐ssTEVp_1–118_‐KDEL‐pA) and SS‐FRB‐ssTEVp_119–245_‐KDEL (P_hCMV_‐SS‐FRB‐ssTEVp_119–245_‐KDEL‐pA) (5 ng each), and different amounts of pMMH10 plasmid, which encodes SEAP‐TSC‐KDEL (P_hCMV_‐SEAP‐TCS‐KDEL‐pA). At 24 h after transfection, the medium was replaced with 30 µL of fresh medium containing either DMSO or rapamycin (100 nm) and 20 µL aliquots were collected for analysis at different time points. Data are presented as means ± s.d. of *n* = 3 biologically independent samples. ^***^ *p* < 0.001.

To explore the effect of intact sec‐TEVp on the secretion dynamics of KDEL‐tagged proteins, we first used SEAP (human placental secreted alkaline phosphatase) as a human model glycoprotein. Co‐transfection of SEAP‐TCS‐KDEL (pMMH10) and sec‐TEVp‐KDEL (pMMH51) encoding vectors into HEK‐293 resulted in a significant increase (18.4‐fold change) in SEAP secretion, compared with cells transfected with SEAP‐TCS‐KDEL alone (**Figure** **1**B), suggesting that sec‐TEVp releases SEAP accumulated in the ER by removing the KDEL retention signal. To make SEAP secretion trigger‐inducible, we utilized the rapamycin‐induced dimerization domains FKBP (FK506 binding protein) and FRB (FKBP‐rapamycin binding protein) to reconstitute an ER‐localized split sec‐TEVp (ERLSS‐TEVp). Therefore, the N‐terminal split sec‐TEVp domain (ssTEVp, 1–118) was N‐terminally fused to FKBP via a flexible (GGGGS)_2_ linker (FKBP‐(GGGS)_2_‐ssTEVp_1–118_), and the C‐terminal sec‐TEVp domain (ssTEVp, 119–245) was N‐terminally fused to FRB (FRB‐ssTEVp_119–245_) (Figure 1C). To direct both fusion proteins into the secretory pathway and retain them in the ER, they were further N‐terminally fused to the calreticulin‐derived secretion signal (SS)^[^ ^20^ ^]^ and C‐terminally with the ER‐retention signal KDEL (SS‐FKBP‐(GGGGS)_2_‐ssTEVp_1–118_‐KDEL, pMMH26; SS‐FRB‐ssTEVp_119–245_‐KDEL, pMMH27) (Figure 1C). Additionally, the protein of interest was C‐terminally fused with a TEVp‐cleavable TCS‐KDEL sequence (SEAP‐TCS‐KDEL, pMMH10; Figure 1C). To confirm that rapamycin can heterodimerize SS‐FKBP‐(GGGGS)_2_‐ssTEVp_1–118_‐KDEL and SS‐FRB‐ssTEVp_119–245_‐KDEL in the ER and so restore ERLSS‐TEVp's TCS‐specific proteolytic activity which removes KDEL and programs accumulated SEAP for rapid release, we co‐transfected human HEK‐293 cells with all RAPID components (pMMH10, pMMH26, and pMMH27) and profiled the resulting SEAP level in the culture supernatant. Indeed, addition of rapamycin significantly increased SEAP secretion within a few minutes, suggesting that RAPID components quickly heterodimerized, restored activity and efficiently released accumulated SEAP from the engineered cells in the presence of rapamycin (Figure 1D).

**Figure 2:**
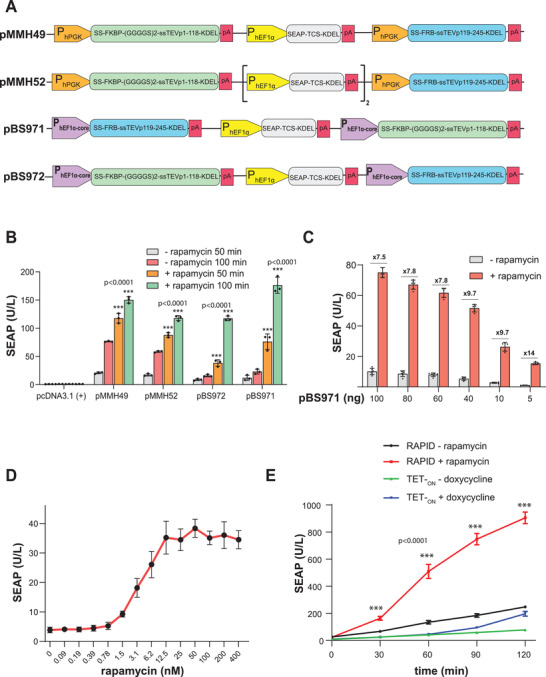
Engineering an efficient RAPID‐based expression system in a single tri‐cistronic expression vector. A) To find the optimal ratios among different parts of RAPID expression system, systematic engineering of different plasmids using P_hEF1a_, P_EF1a core_, and P_hPGK_ was performed. B) SEAP levels in the supernatant of HEK‐293 cells transfected with the different constructs presented previously (100 ng of each plasmid separately). At 24 h after transfection, the medium was replaced with 30 µL of fresh medium containing either DMSO or rapamycin (100 nm) and 20 µL aliquots were collected for analysis at different time points. Data are presented as means ± s.d. of *n* = 3 biologically independent samples. ^***^ *p* < 0.001. C) SEAP level in the supernatant of HEK‐293 cells transfected with different amounts (5–100 ng) of pBS971. 24 h after transfection, the culturing medium was replaced with 30 µL of 100 nm rapamycin‐containing medium (or with DMSO as negative control) and SEAP expression was profiled for after 50 min. Data are presented as means ± s.d. of *n* = 6 biologically independent samples. D) Dose‐response analysis using pBS97 plasmid, which was found to be the most efficient plasmid with minimal leakiness. HEK‐293 cells were transfected with 5 ng of pBS97. At 24 h after transfection, the medium was replaced with 30 µL of fresh medium containing different concentrations of rapamycin and 20 µL aliquots were collected for analysis. Data are presented as means ± s.d. of *n* = 3 biologically independent samples. E) Comparison of RAPID and TET‐_ON_ expression systems. Cells were transfected either with pBS971 or with plasmids encoding P_hCMV*‐1_‐SEAP (P_hCMV*‐1_‐SEAP‐pA, pTS1017) and M2rtTA (P_Ubc_‐M2rtTA‐pA). At 20 h after transfection, the culture medium was replaced with fresh medium and the cells were left for 24 h to recover and to accumulate the system components. The medium was replaced with 30 µL of fresh medium containing either doxycycline (2 µg mL^−1^) or rapamycin (100 nm) and 20 µL aliquots were collected for analysis at different time points. Data are presented as means ± s.d. of *n* = 6 biologically independent samples. ^***^ *p* < 0.001.

To increase transfection and improve the overall system's performance, we cloned all RAPID‐encoding expression units into a single expression vector. To determine the optimal relative expression levels of the RAPID components, we had them driven by constitutive promoters of different strength (P_PGK_, P_hEF1*α*_, and P_hEF1*α*‐core_; **Figure** **2**A). When profiling of rapamycin‐inducible SEAP release in HEK‐293 cells, pBS971 (P_hEF1*α*‐core_‐SS‐FRB‐ssTEVp_119–245_‐KDEL::P_hEF1*α*_‐SEAP‐TCS‐KDEL::P_hEF1*α*‐core_‐SS‐FKBP‐(GGGGS)_2_‐ssTEVp_1–118_‐KDEL) was the best‐in‐class single‐vector RAPID configuration (Figure 2B). Tight and efficient retention of KDEL‐tagged proteins in the ER impacts the overall performance and leakiness of RAPID. To determine the optimal ER‐loading capacity, we transfected human HEK‐293 cells with increasing amounts the RAPID‐expression vector pBS971 (Figure 2C). RAPID's overall rapamycin‐responsive control performance was indeed dependent on the amount of RAPID components (Figure 2C) and could be tuned for optimal control at minimal rapamycin administration (EC_50_ of 10 nm; Figure 2D). To document the rapid protein release dynamics of RAPID we compared its performance to the classical doxycycline‐inducible transcription‐control system.^[^ ^21^ ^]^ RAPID was not only significantly faster in releasing ER‐accumulated SEAP, while TET_ON_‐driven SEAP was delayed by the preceding transcription and translation, the RAPID system also released SEAP in significantly larger amounts (Figure 2E). With its rapamycin‐inducible quick release of large amounts of ER‐accumulated SEAP, RAPID compares dynamically with glucose‐triggered fast vesicular secretion of insulin by *β*‐cells.^[^ ^22^ ^]^

**Figure 3:**
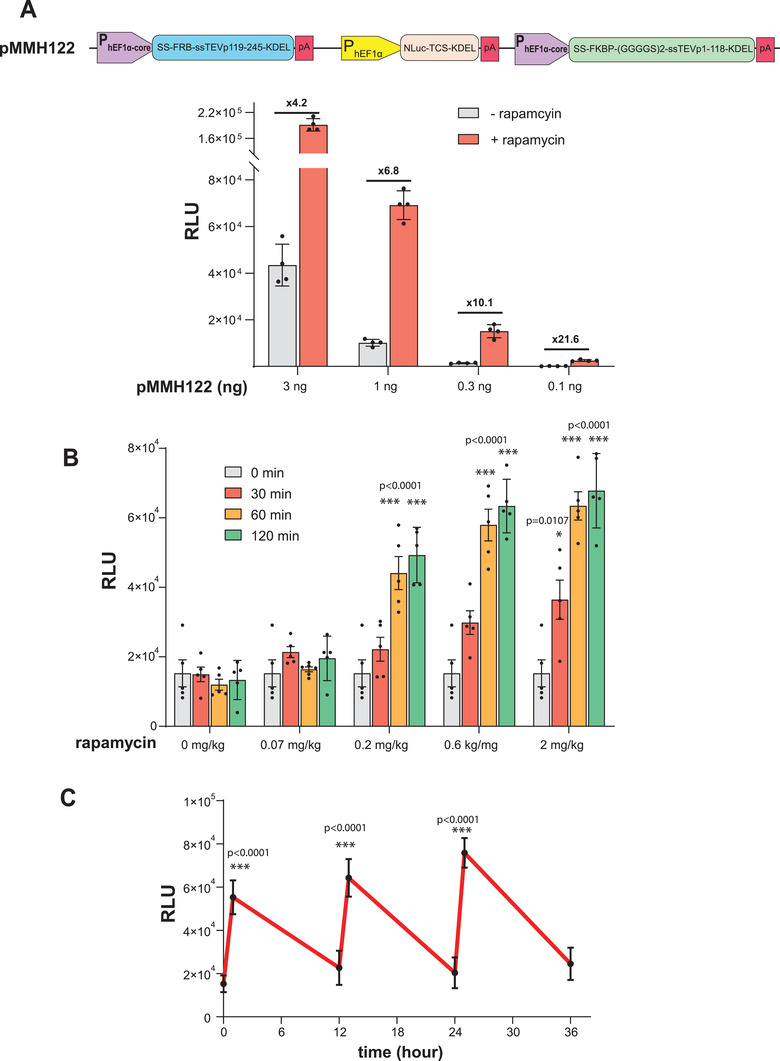
Evaluation of RAPID performance in mice in vivo. A) Top: a schematic illustration of the genetic components of pMMH122, which encodes RAPID‐mediated expression of NanoLuc luciferase (NLuc). Bottom: values of relative luminescence units (RLU) in culture supernatants of HEK‐293 cells transfected with different amounts of pMMH122. At 24 h after transfection, the medium was replaced with 30 µL of fresh medium containing either DMSO or rapamycin (100 nm) and 7.5 µL aliquots were collected for analysis after 50 min. Data are presented as means ± s.d. of *n* = 4 biologically independent samples. B) RLU in the plasma of healthy WT mice following rapamycin treatment at different doses. At 24 h following hydrodynamic transfection of pMMH122, mice were given with different amounts of rapamycin by oral gavage. Blood samples were collected at different time points and RLU values were quantified. Data are presented as means ± s.d. of *n* = 5. C) RLU in the plasma of WT mice following multiple rapamycin treatments. At 24 h following hydrodynamic transfection of pMMH122, mice were treated three times with oral rapamycin (0.2 mg kg^−1^ each time) at 12 h intervals for a period of 36 h. Each point represents the mean of plasma RLU values before or 1 h after rapamycin treatment. Data are presented as means ± s.d. of *n* = 5.

To investigate the performance of the RAPID system in vivo, we chose NanoLuc luciferase (NLuc) as secreted reporter protein, as it has a short half‐life in vivo and is much more sensitive than SEAP reporter protein, thus enabling accurate evaluation of RAPID kinetics using relatively small amounts of blood from the mice. For this purpose, we replaced the SEAP‐TCS‐KDEL expression cassette from pBS971 with NLuc‐TCS‐KDEL to obtain a tricistronic pMMH122 plasmid (**Figure** **3**A top). Before proceeding to in vivo experiments, we first explored the performance of pMMH122 in vitro. HEK‐293 cells were transfected with different amounts of pMMH122 and then exposed to rapamycin (Figure 3A bottom). The addition of rapamycin significantly enhanced NLuc secretion into the supernatant in less than 1 h. In addition, we observed that transfection of small amounts of pMMH122 decreased the basal leakiness of the system and noticeably increased the fold change upon rapamycin addition, a phenomenon that was also observed in the case of SEAP when pBS971 was used (Figure 2C). These results show that RAPID can be utilized for different proteins of interest, and indicate that it may be suitable as a universal platform for fast protein release. The next step was to examine the dose of rapamycin required for RAPID activation in mice. For this purpose, healthy WT mice were hydrodynamically transfected with pMMH122 plasmid through the tail vein. After 24 h, the mice were given either vehicle alone or vehicle containing different concentrations of rapamycin by oral gavage, and blood samples were taken at different time points (Figure 3B). Oral rapamycin at 0.2 mg kg^−1^ was sufficient to activate RAPID in less than 1 h in the mice. The main purpose of this dose‐dependence experiment was to determine the minimal amount of rapamycin needed for RAPID induction so that we could evaluate the reproducibility of the system during repeated cycles of activation. Rapamycin is extensively metabolized in the liver, having an oral bioavailability of around 14%.^[^ ^23^ ^]^ By using a low dose, we could “transiently” expose the liver to a low, but sufficient, concentration of rapamycin for RAPID activation. This low dosage ensures short‐term activation of RAPID, because rapamycin elimination (though liver‐mediated metabolism) reduces the concentration to levels that can no longer stimulate the system, unless a further dose is given. Note that in this model, the liver is both the target and the elimination organ. Here, to examine the reversibility of the system during consecutive activations, mice transfected with pMMH122 plasmid were treated with the minimal effective dose of rapamycin identified above (0.2 mg kg^−1^ orally) three times at 12 h intervals (rapamycin was given by gavage at 0, 12, and 24 h). Blood samples were taken just before and 1 h after each rapamycin treatment (Figure 3C).

**Figure 4:**
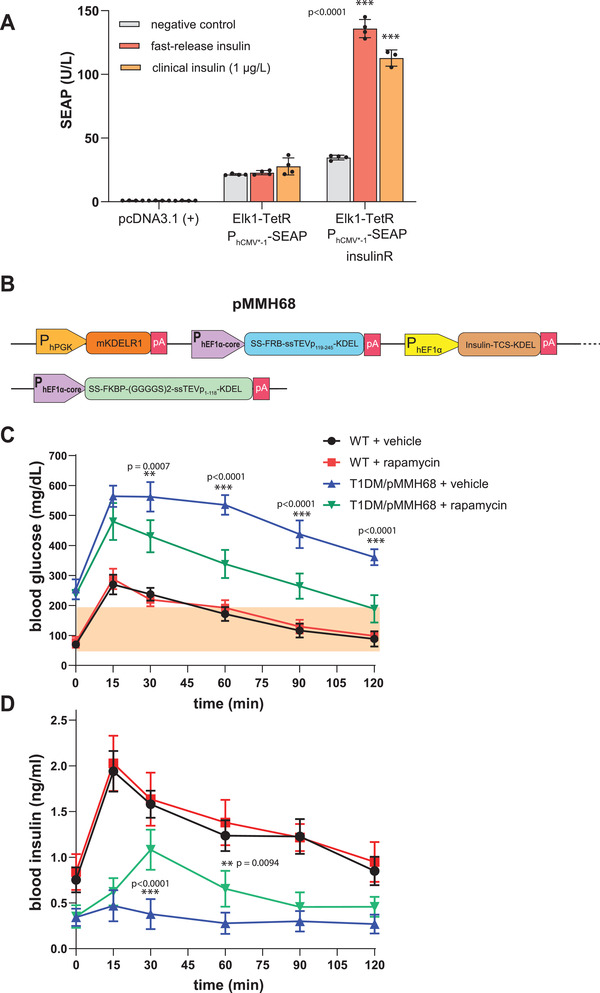
Fast insulin release in T1DM in response to oral administration of rapamycin. A) SEAP levels in insulin‐activity bioassay as a readout of insulin activity. To produce fast‐release insulin, HEK‐293 cells were transfected either with INS‐TCS‐KDEL (pMMH50) in combination with intact sec‐TEVp (pMMH51), or with pcDNA3.1 (+) as a negative control. At 12 h after transfection, the culture medium was replaced with fresh DMEM and incubation was continued for 24 h. The supernatant was used for insulin‐activity bioassay, and SEAP measurements were performed after 24 h (see insulin quantification in the Experimental Section). Clinical insulin (1 µg L^−1^) was used as a positive control. Data are presented as means ± s.d. of *n* = 4 biologically independent samples. ^***^ *p* < 0.001. B) The structures of tetra‐cistronic insulin RAPID‐based expression plasmids used for in vivo hydrodynamic transfection of the liver (pMMH68). C) Mealtime challenge in T1DM or healthy WT mice following rapamycin treatment. At 24 h following hydrodynamic transfection of pMMH68, mice were fasted for 8 h and treated with oral rapamycin gavage (0.2 mg kg^−1^) or vehicle plus to intraperitoneal glucose injection (125 g kg^−1^). Blood glucose levels were then measured at different time points. Normal glycemia values are indicated by an orange band. Data are presented as means ± s.d. of *n* = 5. ^***^ *p* < 0.001. D) Blood insulin profiles in mice from the previous experiment (Figure 3C) using cell culture‐based bioassay. Data are presented as means ± s.d. of *n* = 5. ^***^ *p* < 0.001.

To validate RAPID performance for fast secretion of insulin, we next tested the system for the treatment of experimental type‐1 diabetes. Therefore, we fused TCS‐KDEL to the C‐terminus of insulin's *α* chain (INS‐TCS‐KDEL, pMMH57), so that insulin accumulated in the ER and was rapidly released following administration of rapamycin. RAPID‐released insulin was physiologically active as it binds and activates native insulin receptor (IR) in a cell‐based assay (**Figure** **4**A). For optimal in vivo validation we united all RAPID components in a single expression vector that also contained a constitutive KDELR1^[^ ^24^ ^]^ expression unit (P_hPGK_‐KDELR1) to provide more ER‐anchor points for insulin, which is expected to decreases the overall leakiness of the device^[^ ^24^ ^]^ (pMMH68, P_hPGK_‐KDELR1::P_hEF1*α*‐core_‐SS‐FRB‐ssTEVp_119–245_‐KDEL::P_hEF1*α*_‐INS‐TCS‐KDEL::P_hEF1*α*‐core_‐SS‐FKBP‐(GGGGS)_2_‐ssTEVp_1–118_‐KDEL) (Figure 4B)

To validate the rapid‐release system for the treatment of experimental type‐1 diabetes, we hydrodynamically transfected the liver of type‐1‐diabetic mice with pMMH68 encoding all RAPID components including the retention‐engineered insulin variant. At 24 h after transfection, treated animals were starved for 8 h and received a single oral dose of rapamycin (0.2 mg kg^−1^ through oral gavage) along with intraperitoneal glucose injection (125 g kg^−1^) before their blood glucose (Figure 4C) and insulin levels were profiled (Figure 4D). The RAPID system provided fast insulin release within minutes and attenuated postprandial glycemic excursions in treated mice. WT mice treated with rapamycin alone did not show any effect on blood glucose or insulin levels. These results show that RAPID is able to store sufficient amounts of insulin in the ER and quickly release it in response to a single dose of rapamycin to attenuate glycemic excursions with dynamics similar to that of wild‐type mice.

## 3 Discussion

Fast vesicular release of proteins is an endogenous secretion mechanism common to many endocrine systems including pancreatic blood‐sugar control.^[^ ^25^ ^]^ Vesicular secretion systems produce and store substantial quantities of potent peptide hormones and rapidly release them in response to a trigger, producing a dramatic effect throughout the entire body to a short period of time.^[^ ^26^ ^]^ By contrast, classical trigger‐inducible gene switches considered for cell‐based therapies control transcription, which involves a delay in ramping up production, retards the onset of biopharmaceutical release, and provides slow, continuous low‐level secretion leading to a response that could be too little, too late.^[^ ^27^ ^]^ As shown by direct comparison with the TET_ON_ transcription‐control system, the drug‐inducible store‐and‐release technology of RAPID shows a quicker and higher‐level release pattern that mimics the vesicular secretion of *β*‐cells. Indeed, RAPID‐controlled insulin release restores glucose homeostasis in experimental type‐1 diabetes, providing control similar to that seen in wild‐type mice. Additionally, RAPID provides insulin‐mediated blood‐glucose control by a single oral dose of a clinically licensed drug, which advances the treatment options for diabetes, may improve patient convenience, and could make regular daily insulin injections a thing of the past.

Fast release expression systems are valuable technologies with multiple potential applications, including in urgent pain management. Currently, the main analgesic drugs used are non‐steroidal anti‐inflammatory drugs or narcotic opioids. Despite their wide use, these medications are associated with limited efficacy, serious adverse effects, and issues of drug tolerance and addiction.^[^ ^28^ ^]^ In recent years, various peptide‐based analgesics have been characterized and some, such as ziconotide, have been clinically approved.^[^ ^29^ ^]^ We believe the RAPID system is a promising candidate for rapid delivery of analgesic peptides in order to quickly manage debilitating pain.

Due to its modularity, RAPID can be a flexible platform, since the secreted protein is not limited to SEAP, NLuc, or insulin as employed in the present work, but can be easily and rapidly changed. Furthermore, many chemically induced dimerization systems such as those responsive to caffeine,^[^ ^30^ ^]^ gibberellic,^[^ ^31^ ^]^ or abscisic acid^[^ ^32^ ^]^ are available to expand the oral control input. Fast vesicular secretion of proteins has so far been limited to release of peptide hormones by endocrine cells.^[^ ^33^ ^]^ RAPID extends this quick release feature to non‐endocrine somatic cell types, and is expected to find applications over a broad spectrum of basic, translational, and clinical research. Rapamycin is an FDA‐approved drug that has been in clinical use for decades for various indications, including life‐long immunosuppression to prevent organ rejection after transplantation.^[^ ^34^ ^]^ Since rapamycin has recently also been shown to prevent diabetic complications such as retinopathy^[^ ^35^ ^]^ and to increase insulin sensitivity in type‐1 diabetic patients,^[^^]^ we believe the rapamycin‐triggered RAPID system will be particularly well suited for rapid release of insulinogenic drugs in future cell‐based diabetes therapies.

## 4 Experimental Section

### Chemical and Reagents

Rapamycin (cat. no. 553210‐10MG), doxycycline (cat. no. D9891‐1G), streptozotocin (cat. no. S0130‐500MG), TWEEN 80 (cat. no. P1754‐500ML), and recombinant insulin (catalog no. I0516), were all purchased from Sigma Aldrich (Buchs, Switzerland).

### Plasmid Construction

Gene expression vectors were constructed either by employing restriction endonucleases (New England Biolabs, Ipswich, MA, USA) followed by ligation using T4 DNA ligase (New England Biolabs, cat. no. M0202L) or by Gibson assembly (New England Biolabs, cat. no. E2611L). For restriction enzymes‐based cloning, digested plasmid backbones were dephosphorylated with antarctic phosphatase (New England Biolabs, cat. no. M0289L) before ligation. PCR reactions were performed using either Phusion High‐Fidelity DNA polymerase (New England Biolabs, cat. no. M0530L) or Q5 High‐Fidelity DNA polymerase (New England Biolabs, cat. no. M0491L). For Gibson assembly, the PCR products were amplified using primers that had 15–20 bp complementary sequences at each end. Detailed information about restriction enzymes and primers used for the design of each plasmid is presented in Table S1, Supporting Information. Plasmids were transfected and propagated in XL10‐Gold ultracompetent *Escherichia coli* (New England Biolabs, cat. no. C2992) and DNA was extracted using a plasmid mini‐prep kit (Zymo Research, Irvine, CA, USA, cat. no. D4054) or a ZymoPURE II Plasmid Midiprep Kit (Zymo Research, cat. no. D4200). A PureLink HiPure Expi plasmid megaprep kit (Thermo Fischer Scientific, Waltham, MA, USA, cat. no. K210008XP) was used for larger‐scale preparation.

### Cell Culture

Human embryonic kidney cells (HEK‐293T, ATCC: CRL‐11268) were cultivated in Dulbecco's modified Eagle's medium (DMEM; Thermo Fischer Scientific, cat. no. 10566016) supplemented with 10% fetal bovine serum (Sigma Aldrich, cat. no. F7524, lot no. 022M3395) and penicillin (100 U)‐streptomycin (100 µg) solution (Sigma Aldrich, cat. no. P433) under a humidified atmosphere of 5% CO_2_ in air at 37 °C. Passaging of pre‐confluent HEK‐293 cultures was performed by trypsinization with 0.05% trypsin‐EDTA (Life Technologies, Carlsbad, CA, USA; cat. no. 25300‐054) for 5 min at 37 °C. Cells were transferred to 10 mL cell culture medium, and centrifuged for 1 min at 200 × g. The supernatant was discarded and the cells were resuspended in fresh medium. Cell number and viability were quantified using an electric field multichannel cell‐counting device (Casy Cell Counter and Analyzer Model TT, Roche Diagnostics GmbH, Rotkreuz, Switzerland).

### Transient Transfection

For plasmid transfection in a 96‐well format, HEK‐293 cells were seeded at a density of 50 000 cells per 1 cm^2^ in 100 µL medium for 24 h. 50 µL of serum and antibiotics‐free minimum essential medium MEM (Thermo Fischer Scientific, cat. no. 11095080) containing a 1:5 DNA:PEI mixture (polyethylenimine, MW 40 000; Polysciences Inc., Warrington, FL, USA, cat. no. 24765) with a total DNA amount of 350 ng cm^−2^ was added dropwise to the cells, and the plate was incubated for 12 h.

### SEAP Quantification

SEAP concentration in the cell culture medium was determined as follows: 20 µL of the culture supernatant were mixed with 80 µL ddH_2_O and heat‐inactivated for 30 min at 65 °C. Then, 80 µL of 2× SEAP buffer (20 mm homoarginine, 1 mm MgCl_2_, 21% v/v diethanolamine, pH 9.8) and 20 µL of 120 mm para‐nitrophenyl phosphate (Acros Organics, Geel, Belgium, cat. no. 128860100) solution in 2× SEAP buffer were added to each well, and the absorbance at 405 nm was measured at 37 °C using a Tecan M1000 plate reader (Tecan Group Ltd., Maennedorf, Switzerland). SEAP concentrations were calculated from a standard curve.

### NanoLuc Luciferase Quantification

Blood samples were collected in BD Microtainer blood collection tubes and left for 30 min at room temperature, then centrifuged at 6000 RCF for 5 min, and the plasma were transferred to new tubes. For in vitro experiments, samples were taken directly from the culture supernatants. For luminescence measurement, samples were transferred to black 384‐well plates using 7.5 µL of sample mixed with 7.5 µL working solution of the Nano‐Glo Luciferase Assay System (Promega, catalog number N1130) according the manufacturer's instructions. A Tecan M1000 plate reader (Tecan Group Ltd., Maennedorf, Switzerland) was used to measure luminescence.

### Insulin Quantification

Insulin quantification procedure was performed using a cell culture‐based bioassay. Blood samples were collected into BD Microtainer blood collection tubes and left for 30 min at room temperature. Samples were then centrifuged at 6000 RCF for 5 min, and the plasma were transferred to new tubes and kept at −80 °C until the analysis. For in vitro experiments, culturing media were collected into 1.5 mL Eppendorf tubes, centrifuged at 3000 RCF for 5 min, and the resulted supernatants were isolated for analysis. For insulin quantification, plasma/supernatant samples were added to the insulin‐activity bioassay system, which consisted of HEK‐293 cells expressing IR, and P_hCMV*‐1_‐SEAP, and Elk1‐TetR.^[^ ^38^ ^]^ Once bound to insulin, IR initiated a signaling cascade that ultimately led to activation of the MAPK pathway. The phosphorylation of Elk1‐TetR triggered its translocation to the nucleus, where it bound to P_hCMV*‐1_ promoter, thereby increasing SEAP expression. Insulin concentrations in samples were calculated using a line equation of standard‐curve plot of SEAP levels as a function of standard insulin concentrations.

### Animal Experiments

All experiments involving animals were performed according to the directive of the European Community Council (2010/63/EU), approved by the French Republic (project no. DR2013‐v2), and carried out by Ghislaine Charpin‐El Hamri (license no. 69266309) at the Institut Universitaire de Technologie of the Université Claude Bernard Lyon 1, F‐696226, Villeurbanne Cedex, France.

### Hydrodynamic Transfection of Plasmid DNA into Mice

Mice were placed under a heating lamp for 10 min, then 2 mL of sterile saline solution containing a total of 10 µg DNA was injected through the tail vein within 10 s.^[^ ^39^ ^]^

### Oral Rapamycin Formulation

Rapamycin was first dissolved in DMSO to prepare a stock solution 15 mg mL^−1^. Prior to oral administration, TWEEN‐80 was added at a ratio of 1:5 to the drug solution, and 1:10 dilution was performed with double distilled water. A total of 200 µL of the drug formulation was orally administered per mouse. Negative control mice in this study were given the vehicle, which contained the same ingredients as the drug formulation, but with pure DMSO instead of rapamycin stock solution, by oral gavage.

### Experimental Type‐1 Diabetes

Male C57BL/6JRJ mice purchased from Janvier Labs (Le Genest‐Saint‐Isle, France) were injected with streptozotocin (75 mg kg^−1^ in citrate buffer 0.2 m, pH 4.2) for five consecutive days. Mice were fasted for 8 h before each injection. Type‐1 diabetes was confirmed by measuring glucose levels using a clinically licensed glucometer (Contour Next, Bayer Healthcare, Germany; detection range: 0.5–35 mm), after food restriction for 8 h. WT animals in this study were healthy mice that were not treated with STZ.

### Statistical Analysis

The statistical significance of differences among groups was evaluated with a two‐tailed, unpaired Student's *t*‐test or a one‐way analysis of variance test using GraphPad Prism. Differences were considered statistically significant at *p* < 0.05. The statistical test used and the significance are reported in the figures and figure legends.

## Conflict of Interest

The authors declare no conflict of interest.

## Author Contributions

M.M. and M.F. designed the project, M.M. and B.‐A. S. conducted in vitro experiments, and S.X. and G.C.‐E.H. designed and performed animal experiments. M.M. and M.F. analyzed the data and wrote the manuscript.

## Supporting information

Supporting Information

## Data Availability

The data that support the findings of this study are available from the corresponding author upon reasonable request.
