# Supplementary material for: Engineering a Rapid Insulin Release System Controlled By Oral Drug Administration
Source: Adv Sci (Weinh). 2022 Jan 20;9(9):2105619. doi: 10.1002/advs.202105619 (PMC8948567; doi:10.1002/advs.202105619)
Supplement: Supplementary file 1 — Supporting Information [file ADVS-9-2105619-s001.pdf]

ADVANCED SCIENCE
Open Access

Supporting Information

for *Adv. Sci.*, DOI: 10.1002/advs.202105619

Engineering a Rapid Insulin Release System Controlled By Oral Drug Administration

*Mohamed Mahameed, Shuai Xue, Bozhidar-Adrian Stefanov, Ghislaine Charpin-El Hamri, and Martin Fussenegger**

Table S1

Plasmid	Information and Design	Reference/Source
pcDNA3.1(+)	Mammalian expression vector. (P_{hCMV}-MCS-pA_{bGH})	Life Technologies, Carlsbad, CA
pBS1	Constitutive mammalian SEAP expression vector. ($P_{hEF1\alpha}$-SEAP-pA_{bGH})	Stefanov et al., 2021
pBS717	Tetracycline-inducible mammalian NLuc-mIgGFc expression vector. ($P_{hCMV*-1}$-IgK-NLuc-mIgGFc-pA_{bGH})	Stefanov et al., 2021
pBS828	Tetracycline-inducible mammalian $P_{hCMV*-1}$-driven SEAP and mINS expression vector (ITR-$P_{hCMV*-1}$-SEAP-P2A-mINS-pA_{bGH}:P_{PRPBSA}-EGFP-P2A-ZeoR-pA_{bGH}-ITR)	Stefanov et al., 2021
pBS941	Constitutive mammalian hTRPV1 expression vector. (P_{hPGK}-hTRPV1-pA_{bGH})	Stefanov et al., 2021
pIR	Constitutive human insulin receptor expression vector. (P_{hCMV}-IR-pA_{bGH})	Jacob et al., 2002 Addgene (no.24049)
MKp37	Constitutive mammalian TetR-ELK1 fusion protein expression vector. (P_{hPGK}-TetR-ELK1-pA_{SV40})	Keeley et al. 2018
pTS1017	Tetracycline-responsive SEAP expression vector. ($P_{hCMV*-1}$-SEAP-pA_{bGH}; $P_{hCMV*-1}$, O_{tetO7}-$P_{hCMVmin}$)	Scheller et al., 2020
FUW-M2rtTA	Lentiviral plasmid expressing the reverse tetracycline transactivator. (rtTA)	Hockemeyer et al. , 2008 Addgene (no.20342)
PM-FRB-mRFP-T2A-FKBP-5-ptase	Mammalian expression plasmid used as a template for PCR amplification of FKBP and FRP domains.	Toth et al., 2012 Addgene (no.40896)
pGEM-T KDELRI	cDNA of mouse KDELRI in pGEM-T vector.	SinoBiological (Cat: MG53568-G)
Tier-2	pUC origin of replication based empty backbone for tricistronic mammalian gene expression	Haellman et al., 2021
pBS560	Low-level constitutive promotor for expression of SEAP. The human elongation factor 1-alpha (hEF1a)-core promoter sequence: (*ACGCGT*ctcgaggggcagagcgcacatcgcccacagtccccgagaagttggggggaggggtcggcaattgatccggtgcctagagaaggtggcgcggggtaaactgggaaagtgatgtcgtgtactggctccgcctttttcccgagggtgggggagaaccgtatataagtgcagtagtcgccgtgaacgttctttttcgcaacgggtttgccgccagaacacag*GAATTC*) was synthesised, restricted with *Mlu*I and *EcoR*I and inserted into the corresponding sites (*Mlu*I/*EcoR*I) of pBS1. ($P_{hEF1a\text{-}core}$-SEAP-pA_{bGH})	This work
pBS969	Bi-cistronic mammalian expression vector for production of SEAP-TCS-KDEL and SS-FKBP-$(GGGGS)_2$-ssTEV$p_{1\text{-}118}$-KDEL. $P_{EF1\alpha\text{-}core}$- SS-FKBP-$(GGGGS)_2$-ssTEV$p_{1\text{-}118}$-KDEL was restricted using *Mlu*I and *Hin*dIII from pMMH40 and inserted into the corresponding overhangs in pMMH47 generated through *BsmB*I restriction. ($P_{hEF1\alpha}$-SEAP-TCS-KDEL-pAp_{36} :: $P_{EF1\alpha\text{-}core}$- SS-FKBP-$(GGGGS)_2$-ssTEV$p_{1\text{-}118}$-KDEL -$pA_{p9}$)	This work
pBS970	Bi-cistronic mammalian expression vector for production of SEAP-TCS-KDEL and SS-FRB-ssTEV$p_{119\text{-}245}$-KDEL. $P_{EF1\alpha\text{-}core}$- SS-FRB-ssTEV$p_{119\text{-}245}$-KDEL was restricted using *Mlu*I and *Hin*dIII from pMMH39 and inserted into the corresponding overhangs in pMMH47 generated through *BsmB*I	This work

	restriction. ($P_{hEF1\alpha}$-SEAP-TCS-KDEL-pA_{p36} :: $P_{EF1\alpha\text{-}core}$- SS-FRB-ssTEV$p_{119\text{-}245}$-KDEL -$pA_{p9}$)	
pBS971	Tri-cistronic mammalian expression vector for production of SS-FRB-ssTEV$p_{119\text{-}245}$-KDEL, SEAP-TCS-KDEL, and SS-FKBP-$(GGGGS)_2$-ssTEV$p_{1\text{-}118}$-KDEL. $P_{EF1\alpha\text{-}core}$- SS-FRB-ssTEV$p_{119\text{-}245}$-KDEL was restricted with *Mlu*I and *Hin*dIII from pMMH39 and inserted into the corresponding sites (*Mlu*I/*Hin*dIII) of pBS969. ($P_{EF1\alpha\text{-}core}$- SS-FRB-ssTEV$p_{119\text{-}245}$-KDEL -$pA_{bGH}$ :: $P_{hEF1\alpha}$-SEAP-TCS-KDEL-pA_{p36} ::$P_{EF1\alpha\text{-}core}$- SS-FKBP-$(GGGGS)_2$-ssTEV$p_{1\text{-}118}$-KDEL -$pA_{p9}$)	This work
pBS972	Tri-cistronic mammalian expression vector for production of SS-FKBP-$(GGGGS)_2$-ssTEV$p_{1\text{-}118}$-KDEL, SEAP-TCS-KDEL and SS-FRB-ssTEV$p_{119\text{-}245}$-KDEL. $P_{EF1\alpha\text{-}core}$- SS-FKBP-$(GGGGS)_2$-ssTEV$p_{1\text{-}118}$-KDEL was restricted with *Mlu*I and *Hin*dIII from pMMH40 and inserted into the corresponding sites (*Mlu*I/*Hin*dIII) of pBS970. ($P_{EF1\alpha\text{-}core}$- SS-FKBP-$(GGGGS)_2$-ssTEV$p_{1\text{-}118}$-KDEL-$pA_{bGH}$ :: $P_{hEF1\alpha}$-SEAP-TCS-KDEL-pA_{p36} :: $P_{EF1\alpha\text{-}core}$- SS-FRB-ssTEV$p_{119\text{-}245}$-KDEL -$pA_{p9}$)	This work
pMMH1	Total DNA synthesis of CALsp-sec-TEVp-KDEL by TWIST: atgctgctatccgtgccgctgctgctcggcctcctcggcctggccgtcgcccagggcgcgcaaggggaaagcctgttcaagggaccaagggactacaatccaatctcctcaactatctgccacctgactcaggaaagcgacggacataccacatctctgtacggaattggcttcgggcccttcatcattactaacaagcacctgtttcggagaaacaatggcaccctgctggtgcagagtctgcacggggtgttcaaggtcaaaaatactaccacactgcagcagcatctgattgacggacgagatatgatcattatccggatgccaaaggacttcccccctttccccagaagctgaagttccgggagccccagagggaggaacgcatctgcctggtgactaccaacttccagaccaaatccatgagctccatggtctccgacacctcttctacattcccttctagtgatggcatcttctggaagcactggatccagacaaaagacggacagtgcggcagtccactggtgtcaaccagagatgggtttattgtcggaatccattcagccagcaacttcggaaatactaacaattacttcacctctgtgcccaaaaacttcatggagctgctgactaatcaggaagcacagcagtgggtgagcggatggcgcctgaatgctgattccgtgctgtggggcgggcataaggtcttcatgagcaaacctgaagagccatttcagcccgtcaaggaagccacccagctgatgaacgaagggggcctggaaggcggctctaaggacgagttgtaa	This work (TWIST total synthesis)
pMMH10	Constitutive mammalian SEAP-TCS-KDEL expression vector. SEAP was PCR-amplified from pBS1 and cloned into the CMV-MCS backbone through *Eco*RI and *Hin*dIII restriction enzymes using the following primers: SEAP-*Eco*RI-F (5`-agcggaattcaccatgactagtctgctgctgct) and SEAP-TCS-KDEL-*Hin*dIII-R (5`-cctcaaagctttacaactcgtccttagagccgccggattgaaagtacaggttctcgacaccggtggatccgctagcg). (P_{hCMV}-SEAP-TCS-KDEL-pA_{bGH})	This work
pMMH26	Cloning of this construct was performed through Gibson assembly. First, SS-FKBP-$(GGGGS)_2$ was PCR-amplified from PM-FRB-mRFP-T2A-FKBP-5-ptase (plasmid #40896 addgene) using the following primers: (5`-agctgttcgaagcggaattcatgctgctatccgtgccgctgctgctcggcctcctcggcctggccgtcgccggagtgcaggtggaaaccatctcc-3`) and (5`-ctttccccttgcgcgccctgtgaacctccaccgccagaaccacctccgccttccagttttagaagctccacatc-3`). ssTEV$p_{1\text{-}118}$-KDEL was PCR-amplified from pMMH1 using the following primers: (5`- cagggcgcgcaaggggaaagcctg) and (5`-gcaggccggcctcaaagctttacaactcgtccttagagccgccggtctggaagttggtagtcaccag-3`). (P_{hCMV}- SS-FKBP-$(GGGGS)_2$-ssTEV$p_{1\text{-}118}$-KDEL-pA_{bGH})	This work
pMMH27	Cloning of this construct was performed through Gibson assembly. First, SS-FRB was PCR-amplified from PM-FRB-mRFP-T2A-FKBP-5-ptase (plasmid #40896 addgene) using the following primers:(5`-agctgttcgaagcggaattcatgctgctatccgtgccgctgctgctcggcctcctcggcctggccgtcgccatcctctggcatgagatgtggcat) and (5`-ggagaccatggagctcatggatttctttgagattcgtcggaacacatg-3`) . ssTEV$p_{119\text{-}245}$-KDEL was PCR-amplified from pMMH1 using the following primers: (5`-aaatccatgagctccatggtctcc) and (5`-	This work

	gcaggccggcctcaaagcttttacaactcgtccttagagccgcc). (P_{hCMV}- SS-FRB-ssTEV$p_{119-245}$-KDEL -pA_{bGH})	
pMMH36	Constitutive mammalian SEAP-TCS-KDEL expression vector under hEF1α promoter. SEAP-TCS-KDEL was excised from pMMH10 with *EcoR*I/*Xba*I and inserted into the corresponding sites (*EcoR*I/*Xba*I) of pBS1. ($P_{hEF1\alpha}$-SEAP-TCS-KDEL-pA_{bGH})	This work
pMMH37	Constitutive mammalian expression vector encoding SS-FRB-ssTEV$p_{119-245}$-KDEL under PGK promoter. SS-FRB-ssTEV$p_{119-245}$-KDEL was excised from pMMH27 with *EcoR*I/*Hin*dIII and inserted into the corresponding sites (*EcoR*I/*Hin*dIII) of pBS941. (P_{hPGK} - SS-FRB-ssTEV$p_{119-245}$-KDEL -pA_{bGH})	This work
pMMH38	Constitutive mammalian expression vector encoding SS-FKBP-$(GGGGS)_2$-ssTEVp_{1-118}-KDEL under PGK promoter. SS-FKBP-$(GGGGS)_2$-ssTEVp_{1-118}-KDEL was excised from pMMH26 with *EcoR*I/*Hin*dIII and inserted into the corresponding sites (*EcoR*I/*Hin*dIII) of pBS941. (P_{hPGK} -SS-FKBP-$(GGGGS)_2$-ssTEVp_{1-118}-KDEL-pA_{bGH})	This work
pMMH39	Constitutive mammalian expression vector encoding SS-FRB-ssTEV$p_{119-245}$-KDEL under EF1α_{-core} promoter. SS-FRB-ssTEV$p_{119-245}$-KDEL was excised from pMMH27 with *EcoR*I/*Hin*dIII and inserted into the corresponding sites (*EcoR*I/*Hin*dIII) of pBS560. ($P_{hEF1\alpha-core}$ - SS-FRB-ssTEV$p_{119-245}$-KDEL -pA_{bGH})	This work
pMMH40	Constitutive mammalian expression vector encoding SS-FKBP-$(GGGGS)_2$-ssTEVp_{1-118}-KDEL under EF1α_{-core} promoter. SS-FKBP-$(GGGGS)_2$-ssTEVp_{1-118}-KDEL was excised from pMMH26 with *EcoR*I/*Hin*dIII and inserted into the corresponding sites (*EcoR*I/*Hin*dIII) of pBS560. ($P_{hEF1\alpha-core}$ - SS-FKBP-$(GGGGS)_2$-ssTEVp_{1-118}-KDEL -pA_{bGH})	This work
pMMH47	Constitutive mammalian SEAP expression vector in Tier-2. SEAP-TCS-KDEL was excised from pMMH36 with *Mlu*I/*Hin*dIII and inserted into *Bsa*I-digested Tier-2. ($P_{hEF1\alpha}$-SEAP-TCS-KDEL-pA_{p36}).	This work
pMMH48	Bi-cistronic, constitutive mammalian expression vector of SEAP-TCS-KDEL and SS-FRB-ssTEV$p_{119-245}$-KDEL. SS-FRB-ssTEV$p_{119-245}$-KDEL was excised from pMMH37 with *MluI*/*Hin*dIII and inserted into *Bsmb*I-digested pMMH47. ($P_{hEF1\alpha}$-SEAP-TCS-KDEL-pA_{p36} :: P_{hPGK} - SS-FRB-ssTEV$p_{119-245}$-KDEL -pA_{p9}).	This work
pMMH49	Tri-cistronic, constitutive mammalian expression vector of SS-FKBP-$(GGGGS)_2$-ssTEVp_{1-118}-KDEL, SEAP-TCS-KDEL , and SS-FRB-ssTEV$p_{119-245}$-KDEL. SS-FKBP-$(GGGGS)_2$-ssTEVp_{1-118}-KDEL was excised from pMMH38 with *Mlu*I/*Hin*dIII and inserted into the corresponding sites (*EcoR*I/*Hin*dIII) of pMMH48. (P_{hPGK} - SS-FKBP-$(GGGGS)_2$-ssTEVp_{1-118}-KDEL -pA_{bGH} ::$P_{hEF1\alpha}$-SEAP-TCS-KDEL-pA_{p36} :: P_{hPGK} -SS-FRB-ssTEV$p_{119-245}$-KDEL -pA_{p9}).	This work
pMMH50	Constitutive mammalian expression vector of mINS-TCS-KDEL. mINS was PCR amplified from pBS828 and cloned into pBS1 with TCS and KDEL in the C` terminus using the following primers: mINS-F-*EcoR*I ( 5`-gagaccgaattcatggccctgtggatgcgcttc) and Insulin-TCS-KDEL-*Hin*dIII-R (5`-tcaaagcttttacaactcgtccttagagccgccagagccgccggattgaaagtacaggttctcgttgcagtagttctccagttg). ($P_{hEF1\alpha}$-mINS-TCS-KDEL-pA_{bGH})	This work

pMMH51	Constitutive mammalian expression vector of intact sec-TEVp-KDEL. Intact sec-TEVp was PCR amplified from pMMH1 and cloned into the CMV-MCS backbone through *EcoR*I and *Hin*dIII restriction enzymes using the following primers: sec-TEVp-*EcoR*I-F (5`-gatgatgaattcgccaccatgctgctatccgtg) and sec-TEVp-*Hin*dIII-R (5`-atcatcaagcttttacaactcgtccttag). (P$_{hCMV}$-sec-TEVp-KDEL-pA$_{bGH}$)	This work
pMMH52	Tri-cistronic, constitutive mammalian expression vector of SS-FKBP-(GGGGS)$_2$-ssTEVp$_{1-118}$-KDEL, (SEAP-TCS-KDEL)$_2$, and SS-FRB-ssTEVp$_{119-245}$-KDEL. SEAP-TCS-KDEL cassette was PCR-amplified from pMMH10 and cloned through *Aat*II and *Mlu*I to pMMH49 using the following primers: EF1a-F-*Aat*II (5`-agtgccacctgacgtcgtgcccgtcagtgggcagagcgca) and BGH-R-*MluI* (5`-ggtaccacgcgtccatagagcccaccgcatccccag). (P$_{hPGK}$ -SS-FKBP-(GGGGS)$_2$-ssTEVp$_{1-118}$-KDEL -pA$_{bGH}$ :: (P$_{hEF1\alpha}$ SEAP-TCS-KDEL-pA$_{bGH}$)$_{x2}$:: P$_{hPGK}$ - SS-FRB-ssTEVp$_{119-245}$-KDEL -pA$_{p9}$).	This work
pMMH55	Constitutive mammalian expression vector of SS-FKBP-(GGGGS)$_2$-ssTEVp$_{1-118}$-KDEL in Tier-2. SS-FKBP-(GGGGS)$_2$-ssTEVp$_{1-118}$-KDEL was excised from pMMH39 through *Mlu*I/*Hin*dIII and cloned into *Bsmb*I-digested Tier-2. (P$_{hEF1\alpha\text{-}core}$ - SS-FKBP-(GGGGS)$_2$-ssTEVp$_{1-118}$-KDEL-pA$_{p9}$)	This work
pMMH56	Bi-cistronic, constitutive mammalian expression vector of mINS-TCS-KDEL and SS-FKBP-(GGGGS)$_2$-ssTEVp$_{1-118}$-KDEL in Tier-2. mINS-TCS-KDEL was excised from pMMH50 through *Mlu*I/*Hin*dIII and cloned into *Bsa*I-digested pMMH55. (P$_{hEF1\alpha}$-mINS-TCS-KDEL-pA$_{p36}$ :: P$_{hEF1\alpha\text{-}core}$ - SS-FKBP-(GGGGS)$_2$-ssTEVp$_{1-118}$-KDEL-pA$_{p9}$	This work
pMMH57	Tri-cistronic, constitutive mammalian expression vector of SS-FRB-ssTEVp$_{119-245}$-KDEL, mINS-TCS-KDEL, and SS-FKBP-(GGGGS)$_2$-ssTEVp$_{1-118}$-KDEL in Tier-2. SS-FRB-ssTEVp$_{119-245}$-KDEL was excised from pMMH40 through *Mlu*I/*Hin*dIII and cloned into *Mlu*I/*Hin*dIII-digested pMMH56. (P$_{hEF1\alpha\text{-}core}$ - SS-FRB-ssTEVp$_{119-245}$-KDEL -pA$_{bGH}$ ::P$_{hEF1\alpha}$-mINS-TCS-KDEL-pAp36$_{p36}$ :: P$_{hEF1\alpha\text{-}core}$ SS-FKBP-(GGGGS)$_2$-ssTEVp$_{1-118}$-KDEL -pA$_{p9}$	This work
pMMH66	Constitutive mammalian expression vector encoding mKDELRI under PGK promoter. mKDELRI was PCR-amplified from pGEM-T KDELRI and cloned into pBS941 though *EcoR*I and *Hin*dIII using the following primers: (5`- agaccgaattcatgaacctcttccgattcctg) and (5`-gcctcaaagcttttatgccggcaagctcagctt). (P$_{hPGK}$ -mKDELRI-pA$_{bGH}$)	This work
pMMH68	Tetra-cistronic constitutive mammalian expression vector of mKDELRI, SS-FRB-ssTEVp$_{119-245}$-KDEL, mINS-TCS-KDEL, and SS-FKBP-(GGGGS)$_2$-ssTEVp$_{1-118}$-KDEL. mKDELRI encoding cassette was PCR-amplified and cloned by Gibson assembly into *Mlu*I-linearized pMMH57 using the following primers : hPKG- mKDELR -F (5`-aagaaaccaagatctccacgcgctaccgggtaggggaggcgctttt) and BGH -mKDELR -R (5`- gcgctctgcccctcgagacgcgccatagagcccaccgcatcccc). (P$_{hPGK}$-mKDELRI-pA$_{bGH}$ :: P$_{EF1\alpha\text{-}core}$- SS-FRB-ssTEVp$_{119-245}$-KDEL -pA$_{bGH}$:: P$_{hEF1\alpha}$-mINS-TCS-KDEL-pA$_{p36}$ :: P$_{EF1\alpha\text{-}core}$- SS-FKBP-(GGGGS)$_2$-ssTEVp$_{1-118}$-KDEL-pA$_{p9}$)	This work

pMMH91	Constitutive mammalian expression vector encoding NLuc-TCS-KDEL under P_{hCMV}. NLuc-TCS-KDEL was PCR-amplified from pBS717 using the following primers: NLuc-TCS-KDEL-R: 5`-caagctgTTCGAAGCGGAATTCACCATG and NLuc-TCS-KDEL-R: 5`-cctgcaggccggcctcaaagcttttacaactcgtccttagagccgccagagccgccggattgaaagtacaggttctcagagccgcccgccagaatgcgttcgcacagccgccagccggt.<br>The PCR product was cloned into pcDNA3.1 (+) using Gibson assembly.<br>(P_{hCMV} -NLuc-TCS-KDEL-pA_{bGH}).	This work
pMMH122	Tri-cistronic mammalian expression vector for production of SS-FRB-$ssTEVp_{119-245}$-KDEL, NLuc-TCS-KDEL, and SS-FKBP-$(GGGGS)_2$-$ssTEVp_{1-118}$-KDEL. PCR of whole pBS971 except SEAP-TCS-KDEL was performed using the following primers: RAPID-F 5`- agctatacaggtgcaccgctttataataaa and RAPID-R 5`- gaattctcacgacacctgaaatggaagaaa.<br>NLuc-TCS-KDEL was PCR-amplified from pMMH91 using the following primers: NLuc-KDEL-F 5`-atttcaggtgtcgtgagaattcaccatgactagtgagacagacacactcctg NLuc-KDEL-R 5`- aaagcggtgcacctgtatagctttacaactcgtccttagagccgccagagcc.<br>The two fragments were then ligated using Gibson assembly.<br>($P_{EF1\alpha\text{-}core}$- SS-FRB-$ssTEVp_{119-245}$-KDEL -$pA_{bGH}$ :: $P_{hEF1\alpha}$-NLuc-TCS-KDEL-pA_{p36} ::$P_{EF1\alpha\text{-}core}$- SS-FKBP-$(GGGGS)_2$-$ssTEVp_{1-118}$-KDEL -$pA_{p9}$).	This work

Abbreviations: **EGFP**, enhanced green-fluorescent protein; **ELKI**, ETS transcription factor ELK1; **FKBP**, FK506 binding protein ; **FRB**, FKBP-rapamycin binding; I**gK,** light-chain kappa-derived secretion signal; **IR**, insulin receptor; **ITR** inverted terminal repeats of SB100X; **KDEL**, Lysine, Aspartic acid, Glutamic acid, Leucine; **MCS**, multiple cloning site; **mIgGFc,** murine immunoglobulin gamma heavy chain constant region; **mINS**, optimized insulin variant for expression in HEK-293 cells; **mKDELRI**, mouse KDEL receptor I; **NLuc**, NanoLuc luciferase; **O_{tetO7}**, heptameric TetR-specific operator; **P2A** picornavirus-derived ribosome skipping sequence optimized for bicistronic expression in mammalian cell; **pA_{bGH}**, polyadenylation signal from the bovine growth hormone; **pA_{SV40}**, polyadenylation signal from simian virus 40; **PCR** polymerase chain reaction; **$P_{hEF1\alpha}$**, human elongation factor 1-alpha promoter; **P_{hCMV}**, human cytomegalovirus immediate early promoter; **$P_{hCMVmin}$**, minimal version of P_{hCMV}; **$P_{hCMV*-1}$**, tetracycline-responsive promoter; **P_{hPGK}**, human 3-phosphoglycerate kinase promoter; **P_{RPBSA}**, strong synthetic promoter; **p9/p36**,synthetic polyadenylation sites (Haellman et al., 2021); **SEAP**, human placental secreted alkaline phosphatase; **sec-TEVp**; secretory tobacco etch virus protease; **SS-** secretion signal; **ss-TEVp**, split secretory tobacco etch virus protease; **TCS**, TEVp cleavage site; **TetR**, *Escherichia coli Tn*10-derived tetracycline-dependent repressor; **tTA**, tetracycline-dependent transactivator (TetR-VP16); **rTetR**, reverse TetR; **rtTA**, reverse tetracycline-dependent transactivator (rTetR-VP16); **TRPV1**, transient receptor potential vanilloid 1; **ZeoR**, zeocin resistance gene.

Oligonucleotides: sequences specific for restriction endonucleases and Gibson assembly overlapping sequences are underlined.

References

Haellman, V., Strittmatter, T., Bertschi, A., Stücheli, P. & Fussenegger, M. A versatile plasmid architecture for mammalian synthetic biology (VAMSyB). *Metab. Eng.* **66**, 41–50 (2021).

Hockemeyer, D. *et al.* A Drug-Inducible System for Direct Reprogramming of Human Somatic Cells to Pluripotency. *Cell Stem Cell* **3**, 346–353 (2008).

Jacob, K. K., Whittaker, J. & Stanley, F. M. Insulin receptor tyrosine kinase activity and phosphorylation of tyrosines 1162 and 1163 are required for insulin-increased prolactin gene expression. *Mol. Cell. Endocrinol.* **186**, 7–16 (2002).

Keeley, M. B., Busch, J., Singh, R. & Abel, T. TetR hybrid transcription factors report cell signaling and are inhibited by doxycycline. *Biotechniques* **39**, 529–535 (2005).

Scheller, L. *et al.* Phosphoregulated orthogonal signal transduction in mammalian cells. *Nat. Commun.* **11**, (2020).

Stefanov, B. A. *et al.* Genetically Encoded Protein Thermometer Enables Precise Electrothermal Control of Transgene Expression. *Adv. Sci.* **2101813**, 1–12 (2021).

Tóth, D. J. *et al.* Acute depletion of plasma membrane phosphatidylinositol 4,5-bisphosphate impairs specific steps in endocytosis of the G-protein-coupled receptor. *J. Cell Sci.* **125**, 3013 (2012).
